# Nutritional Considerations for Performance in Young Athletes

**DOI:** 10.1155/2015/734649

**Published:** 2015-08-19

**Authors:** JohnEric W. Smith, Megan E. Holmes, Matthew J. McAllister

**Affiliations:** Department of Kinesiology, Mississippi State University, P.O. Box 6186, Mississippi State, MS 39762, USA

## Abstract

Nutrition is an integral component to any athletes training and performance program. In adults the balance between energy intake and energy demands is crucial in training, recovery, and performance. In young athletes the demands for training and performance remain but should be a secondary focus behind the demands associated with maintaining the proper growth and maturation. Research interventions imposing significant physiological loads and diet manipulation are limited in youth due to the ethical considerations related to potential negative impacts on the growth and maturation processes associated with younger individuals. This necessary limitation results in practitioners providing nutritional guidance to young athletes to rely on exercise nutrition recommendations intended for adults. While many of the recommendations can appropriately be repurposed for the younger athlete attention needs to be taken towards the differences in metabolic needs and physiological differences.

## 1. Introduction

Current estimates suggest approximately 35 million youth between the ages of 5–18 years participate in organized sports each year [1]. While a majority of these young athletes are playing sports for the aspects of comradery and fun, a growing segment of young athletes train to enhance their opportunity to make a career of sport. While elite sport has long seen the presence of young athletes (Nadia Comaneci, 14 years of age (1976 Olympic Gold Medalist), Marjorie Gestring, 13 years of age (1936 Olympic Gold Medalist), and Dimitrios Loundras, 10 years of age (1896 Olympic Bronze Medalist)), the past few decades have experienced an expansion in the numbers of young athletes working to perform at higher levels as younger athletes. This expansion can be seen in the establishment of the many facilities open focusing specialized training for sports performance on not only elite athletes but also largely youth athletes.

While the increase in physical activity of youth is important we currently do not fully understand the effects such training has on the growth and development of youth. The American Academy of Pediatrics outlined potential risks associated with sports specialization in young athletes in a publication in 2000 [2]. Noted orthopedic surgeon, Dr. James Andrews, recently discussed the potential for negative effects of specialized training on developing bodies and the rise in youth sport injuries he experienced since around the same 2000 timeframe [3]. It is not within the scope of this review to discuss the ethical considerations of having youth focus their training on a singular sport, nor to discuss the potential for injury as related to overuse injuries. However, with the continual trend in younger athletes training for high level performance it appears that our current options are to continue to underscore the potential risks while at the same time work with the participants providing as much assistance as possible to enhance safety.

Proper nutrition is a fundamental component of athletes' training and performance plan. Proper nutrition ensures that an individual is amassing the fuels necessary for the energy production needs related to activity and recovery. One of the areas needing to be addressed is the unique nutritional needs associated with intense exercise stress. However, our understanding of the effects of strenuous physiological training and nutritional variations in combination with exercise stress in youth athletes is greatly limited. This limited knowledge is most likely due to the ethical considerations of withholding nutrients and physiologically overstressing a vulnerable population such as children and adolescents still in the process of growth and development.

Our knowledge regarding the nutritional needs of youth is based on the needs related to proper growth and development in healthy children or those suffering from illness. Most of the knowledge we possess related to the physiological adaptations to training, exercise performance, and sports nutrition is based on research conducted in college aged, middle aged, and older adult populations. Therefore, most sports nutrition recommendations promoted in youth sport are actually based on findings in adult populations. While this is a starting point, research has shown that adolescent energy expenditure and metabolism can differ from those of their adult counterparts so many of these recommendations may not provide ideal insight into the nutritional needs of the youth athlete [4–6].

The goal of this review is to compile an overview of our understanding of the nutritional needs of the young athlete during training and competition. We will also identify the knowledge gaps that currently exist in our understanding of this vulnerable population's needs around physiologically stressing occasions. Due to the limited research on the young athlete population, in many instances the knowledge gained through research on adult populations is the only means to provide recommendations for the young athlete.

Nutrition for healthy growth and maturation is governed by a variety of parameters, each essential in the development from child to adult. This paper emphasizes the importance of adolescent nutrition by first examining gross total caloric intake to better understand the energy requirements of adolescents. Total caloric intake must be sufficient to meet the additional demands of growth, which vary at different stages of growth and maturation and between individual children. Likewise, the proportion of calories allocated to each macronutrient is heavily dependent on the situational constraints of the individual child, which is further complicated by the physiological constraints of a given level of development. This paper emphasizes the importance of each macronutrient with specific focus on the physiological nuances of adolescent metabolism specifically focused around the young athlete. Similarly, micronutrient needs are driven by demands of growth and maturation as well as activity levels. Unique demands of the growing adolescent have highlighted a few select micronutrients in the literature which will be reviewed here following a general overview of micronutrient needs.

## 2. Growth and Development

Growth, maturation, and development are three constructs paramount in any discussion regarding youth. While these terms often manifest concurrently in youth, they refer to three different parameters. Growth simply refers to the quantifiable increase in size, whereas maturation refers to timing and tempo of progress toward the mature state. Timing and tempo refer to the age at which specific maturational events occur and rate at which an individual progresses through these events. Both timing and tempo vary considerably between children [8]. Development is considered a social construct that typically focuses on behaviors and attitudes. Behaviors and attitudes developed during childhood and adolescence provide the basis for adult behaviors and attitudes. Refinement of accepted behaviors in a society requires the development of competencies in an array of interrelated domains that ultimately shape a given behavior and attitude toward that behavior. Taken together, growth, maturation, and development synergistically influence an individual's general self-concept and self-esteem [8]. This holistic perspective is often overlooked when focused on specific pediatric topics, such as nutrition.

Much like business, calorie supply (i.e., energy intake) is dictated by demand (i.e., energy expenditure). Energy expenditure is represented by four major components in children and adolescents: basal/resting metabolic rate, thermic effect of food, thermic effect of activity, and the energy requirements of growth [8]. Basal and resting metabolic rates (BMR and RMR, resp.) vary chiefly on assessment methodology, but only marginally in amount of calories. For the purposes of this discussion, the term RMR will be used to represent both. In adults RMR increases proportionally with body mass, particularly lean body mass [9]. Similarly, RMR increases as children gain body mass. However, when RMR is examined per unit of body mass, RMR decreases as children progress to their adult size [10], which demonstrates the contribution of growth to RMR in children and adolescents. The thermic effect of food varies significantly by the proportion of macronutrients comprising the food consumed. On average, 6–8% of ingested calories are used in the digestive, absorptive, and storage processes of food. Thermic effect of activity is the most variable component of energy expenditure and refers to the calorie cost of movement. When estimating caloric requirements, activity levels are examined at three levels: light, moderate, and vigorous lifestyle physical activity. Given the significant participation in high energy demanding activities, vigorous lifestyle physical activity is exemplified in the youth athlete population. The energy cost of growth is examined in two parameters, the energy to synthesize tissue and the energy deposited in those tissues [7]. Growth varies according to the tempo of maturational development and is very rapid during infancy and early childhood and, thus, accounts for a greater proportion of caloric expenditure. Conversely, during late childhood and adolescence, growth accounts for 1-2%, which reflects a slower rate of growth [8]. With consideration to each of these four components, the FAO/WHO/UNU expert panel used typical weight gains per year to develop age specific and gender specific caloric recommendations [7].  Table 1 shows the caloric recommendations for boys and girls participating in vigorous lifestyles physical activity. Daily energy requirements increase with age and are similar between boys and girls until pubertal ages.

## 3. Protein

Protein is needed for normal cellular functioning as well as synthesis of various bodily tissues [11]. Athletes tend to have elevated demands for dietary protein intake compared to sedentary individuals [12]. As a general recommendation for maintaining health, current recommendations are between 0.8 and 1.2 grams of protein per kg of body mass daily [13]. This recommendation is sufficient to meet the bodily demands of 97.5% of the population, which also accounts for variations in demographic BMI as well as gender [11]. A review by Nemet and Eliakim speculates these requirements are likely sufficient for children and youth athletes. The American College of Sports Medicine and American Dietetic Association recommend intakes between 1.2 and 1.8 g/kg of body mass for active adults [14, 15], which appears to be an adequate requirement for youth athletes [16, 17]. Protein synthesis is highest during infancy and, as such, during this time relative dietary protein intake is at an elevated demand [11]. The question of how much dietary protein is needed to maximize performance among athletes is a question that has been debated for more than 150 years [11, 18] and still remains a debate. Recent evidence suggests two to three times the RDA for protein intake may be optimal to enhance fat-free mass during periods of caloric restriction [19] which may be commonly practiced among athletic populations to achieve a body composition more favorable for performance. Investigation of dietary intakes for various youth age groups suggests that intakes this high are often achieved in normal dietary patterns [20], which indicates intake is sufficient to meet the elevated demands.

Many athletes make dietary modifications in attempt to maximize performance and meet body weight requirements for competitive classes [21]. Several studies have shown increased dietary protein intake accompanied by exercise intervention may aid in weight loss as well as preservation of lean body mass typically associated with reduced body weight [22–25]. Some suggest the mechanism may be partially attributed to increased thermogenesis and satiety associated with elevated protein intake. When compared to fat and carbohydrate, protein has a greater thermic effect that is likely only significant enough to result in weight loss when the high protein diet is maintained over the course of several months [26]. Additional research is needed to fully investigate this hypothesis. Several studies have demonstrated satiating effects of high protein diets [27–29], which may better elucidate a mechanism of weight loss with this dietary intervention. Branched chain amino acids found in protein-rich foods are known to assist in preservation of lean body mass [30], which has significant implications for athletes particularly during periods of weight loss. Leucine specifically is one branched chain amino acid that is strongly associated with protein synthesis [31]. This amino acid can be ingested in supplement form; however, when determining the safe tolerable upper intake level for leucine intake, trials in youth are limited. One study suggests the upper level for young males aged 20–35 years is 500 mg/kg/day or 35 g/day based on plasma and urinary ammonia and leucine concentrations [32]. This recommendation has not been examined in youth and caution of leucine at these high levels is warranted. However, food sources such as egg whites and dairy sources contain multiple amino acids and, as such, protein-rich foods should be emphasized to a greater extent than single amino acids alone.

The most significant question is whether or not youth athletes are obtaining the amount of protein needed for their elevated demands. It has been documented that youth athletes in general are achieving protein intakes much greater than the RDA [20]. Given this evidence, it is unlikely beneficial to promote increased protein intake in youth. A thorough dietary evaluation is suggested before promoting increased protein intake in youth athletes. However, as a general recommendation, athletes should ingest balanced protein feedings throughout the day [19] and emphasize whole foods as opposed to protein based supplements due to the lack of scientific support for protein based supplements in comparison to protein-rich whole foods [33].

As previously mentioned, athletes require higher protein intake to maintain protein synthesis [33]. Additionally, research has shown the ingestion of 20 g of protein following exercise helps maintain positive protein balance following exercise [34]. Evidence suggests that protein based supplements are not required to meet this increased demand [2]. Nonetheless, protein supplements remain one of the most common dietary supplements [35] purchased by athletes who seek to increase markers of performance such as speed, strength, power, and hypertrophy [36]. Several reports have documented athletes' perception that protein supplements are necessary to build muscle [37, 38] and achieve peak performance [39]. This notion has been well investigated in adults but also appears true in the limited research regarding youth athletes, specifically high school football players [33]. This misconception among youth is partially driven by the lack of formal knowledge in nutrition [40]. Youth athletes gain a significant amount of nutrition information from magazines, family members, and coaches [41] and, thus, may not be able to make appropriate, evidence based decisions regarding the use of protein supplements [33]. Considering the lack of scientific support for protein based supplements being superior to natural protein containing foods, youth athletes should be advised to consume their protein from whole foods as opposed to supplements.

## 4. Fat

Dietary lipids are essential for the absorption of vitamins A, D, E, and K, as well as synthesis of cholesterol and other sex hormones [42]. In terms of caloric requirements, most sources recommend lipid intake should be limited to 25–30% of total caloric intake [43], which is relatively the same for both sedentary and active individuals. It is important to consider caloric demands are increased in athletic populations; therefore,* absolute* lipid intakes are likely to be higher. The average adolescent consumes roughly one-third of their dietary intake as lipids [44]. It is important to restrict lipid intake to avoid excessive caloric intake; however, there is no health benefit in diets with less than 15% of calories from lipids [2]. In terms of athletic requirements, an increase of dietary carbohydrate should account for a majority of the increased caloric demands, rather than an increase of dietary lipid. Adequate calorie consumption to support periods of rapid growth is of greatest concern when considering nutrition to maximize performance of adolescent athletes [45]. Roughly fifty percent of adult body mass as well as skeletal mass is achieved during puberty. Large increases in lean and adipose tissue are also seen in males and females during the transition from child to adult as well [44]. During this time, dietary fat is especially important to aid in the synthesis of hormones and assist in normal bodily functioning as well as healthy growth and maturation [46]. Dietary lipid intakes beyond 30% are not advised since this could contribute to excessive weight gain [47]. However, acutely, excessive lipid intake can also result in postprandial oxidative stress, which is associated with impaired vascular and metabolic functioning [48, 49]. Elevated lipid intake is also potentially associated with the pathogenesis of cardiovascular disease [42] which is particularly relevant for youth athletes, given that the origins of CVD begin at an early age and progress into adulthood [48, 50, 51]. The organized group setting provides an ideal platform for discussion of nutrition and physical activity habits among individuals who already acknowledge their value.

Adolescents are more efficient in terms of substrate utilization, which has been shown both at rest and during graded exercise tests since younger children derive a higher percentage of energy from lipids as indicated by lower RER values at submaximal intensities [52]. Improved aerobic efficiency is related to increased dependency on lipids for ATP production commonly noted in youth [5]. This could potentially be the result of an adaptive response since infants and toddlers (under the age of 2) require a higher percentage of energy from lipids to support their increased caloric and growth demands [53]. Alterations in dietary lipid intake could contribute to changes of enzymatic activity as well as elevated lipid metabolism [54]. A lack of glycolytic enzyme activity could be another reason for the aforementioned increased dependency on lipid metabolism [4]. During exercise, carbohydrates and lipids are the main sources of skeletal muscle ATP production, with lipids serving as an important source of energy during low and moderate intensity [45]. Chronic exercise training results in favorable mitochondrial adaptations in adults, which favor enhanced lipid metabolism as well [55].

Upon investigation of differences in lipid oxidation among different age and gender groups in children, a review by Aucouturier et al. [4] reported only miniscule differences between age groups among male and female adolescents. These miniscule differences are likely associated with a change in body size (e.g., acquisition of skeletal and muscle mass) during periods of growth and maturation among different age groups and are more significant in males compared to females. However, Aucouturier et al. reported all children (in general) depend more readily on lipids in comparison to adults. This metabolic characteristic could depend on pubertal status, since it has been shown that 12-year-old females demonstrate elevated lipid metabolism during exercise performed at 70% ${\dot{V}\text{O}}_{2\;\max}$ compared to 14-year-old females [56]. Similar findings have also been reported in boys aged 12 and 14 [57]. However, to our knowledge there is little to no evidence showing prepubescent males and females differ* significantly* in terms of relative fat and carbohydrate oxidation during submaximal exercise [4]. In comparison to adults, however, children lack the ability to sustain longer duration exercise, which may be related to a lack of the ability to store glycogen in children [58]. Generalizing substrate utilization during* prolonged* exercise is difficult given the paucity of experimental or quasi-experimental evidence which examines exercise testing in children for greater than one hour in duration [58], which is likely reflective of the general short and intermittent physical activity patterns and behaviors of that age group [59].

The composition of dietary fatty acids (e.g., chain length) can affect fat oxidation during submaximal exercise [54]. However, this response may vary among maturational levels of the young athlete since prepubescent males tend to have a higher percentage of fatty acid oxidation [60]. It is also important to consider the potential adaptations resulting from modifications of lipid intake. Short term elevations in lipid intake are likely to result in positive energy balance which may not be immediately matched with an increase in beta-oxidation [54]. However, trials in adult populations show that exercise can enhance lipid metabolism by stimulating mitochondrial biogenesis [61] as well as increasing activity of lipoprotein lipase [62] and carnitine palmitoyltransferase 1 [63]. The aforementioned adaptations can enhance lipid metabolism and contribute to an accommodated energy balance due to changes in fat metabolism [54]. This evidence is especially applicable to athletes since exercise training has been shown to accommodate the metabolic effects of short term high fat diets [64]. These adaptations, however, still do not constitute promotion of lipid intakes greater than 30% in youth athletes.

As mentioned earlier, factors such as the type of lipid ingested (i.e., composition of the hydrocarbon chain) can affect the subsequent metabolism [54] and potential storage [65, 66]. Piers et al. [67] demonstrated the substitution of saturated fat with monounsaturated fatty acids can potentially have a favorable effect on body composition. The contribution of saturated fatty acid intake to the development of CVD is important [42]; however, a meta-analysis by Siri-Tarino et al. [68] included twenty-one studies and reported no significant risk of coronary heart disease with elevated saturated fatty acid intake. It is important to note coronary heart disease is a* chronic*,* progressive* disease, the origins of which begin early in life. Thus, youth require appropriate dietary advice that may potentially become healthy behaviors in adulthood as a means of preventing or reducing disease risk. Further, these findings [68] may not be applicable to all individuals since intensity of activity and lipid composition both affect lipid metabolism [54]. Considering the evidence to date, dietary unsaturated fatty acids should not serve as exclusively the sole source of lipids. However, as a general recommendation, and in the interest of promoting long-term healthy behaviors, unsaturated fatty acids should be emphasized to a greater extent.

Some athletes may believe certain lipid based supplements may allow for an ergogenic effect given the limited findings suggesting supplementation may enhance lipid metabolism by decreasing dependency on glycogen/glucose for energy metabolism [69]. Commercial marketing is based on the premise some lipid based dietary supplements can thereby increase aerobic capacity and performance, improve lipid metabolism, and reduce inflammatory damage [70]. Fish oil and conjugated linoleic acid (CLA) are two lipid based supplements that have been investigated in relation to potential ergogenic effects.

Fish oil contains two essential fatty acids (eicosapentaenoic acid [EPA] and docosahexaenoic acid [DHA]). Increased dietary intake of these essential fatty acids has been associated with decreased prevalence of cardiovascular diseases [71, 72], as well as reduced markers of inflammation [73, 74]. In regard to physical performance, a variety of trials failed to demonstrate an ergogenic effect of EPA and DHA [75–77]; Tartibian et al. [78] reported improved pulmonary functioning in young wrestlers [78]. In terms of the ability to improve athletic performance, the majority of data demonstrate a lack in ergogenic effect of fish oil ingestion on athletic performance in well trained athletes [76, 79, 80]. The overwhelming scientific support for fish oil supplementation highlights improvements in cardiovascular health and decreases markers of inflammation which* could* contribute to decreased recovery time between exercises as speculated by Macaluso et al. [69]. However, this has not yet been supported in literature. Furthermore, the majority of clinical trials utilize extremely high doses (>3 g/day) [69, 77, 81], which is difficult to achieve without dietary supplementation of fish oil.

Other benefits of fish oil consumption have been noted including improved cognitive function and reduced ADHD symptoms in children [82]. A meta-analysis by Yang et al. [83] indicated dietary fish and/or fish oil supplementation is also associated with a reduced prevalence of asthma in children [83]. However, additional trials in youth (specifically with athletic samples) are warranted as this population has not been investigated to our knowledge. Given the gap in the literature examining youth athletes, clear recommendations for fish oil consumption cannot be made and warrant further investigation.

CLA is another lipid based dietary supplement that has been proposed to improve athletic performance [69]. CLA is found naturally occurring in beef, lamb, and dairy products such as milk and cheese [84] but is also available in supplement form. Animal studies utilizing CLA administration have demonstrated potentially favorable effects on body composition [85]. However, this may not be applicable to humans since Zambell et al. [86] failed to show a change in energy expenditure and lipid metabolism. A recent review by Macaluso et al. [69] reported fish oil and CLA supplementation can* potentially* have a favorable effect on anabolic effects of exercise which could be related to increased testosterone synthesis. However, given the strong relationship between growth, maturation, and anabolic hormone levels in youth, the demands for youth athletes to intentionally manipulate hormone levels are not advised.

## 5. Carbohydrate

Human metabolism relies primarily on the oxidation of fats and carbohydrates as its fuel sources. As physiological intensity increases from rest to vigorous there is a transition from fat functioning as the primary fuel source to carbohydrate supplying a majority of the body's fuel for energy. The sources of carbohydrate for metabolism are glycogen stores in the muscle, glycogen stores in the liver, and exogenous carbohydrate entering the blood stream through the ingestion of carbohydrate. Some confusion exists in athletes understanding of the specific carbohydrate needs and it has been suggested by Burke et al. to stem from the fact that many recommendations are based on percentage of total caloric intake which adds to the difficulty in understanding the dietary needs of carbohydrate in athletes that have caloric intakes which often exceed general recommendations [87].

General carbohydrate intake recommendations suggest adult athletes consume 5–12 grams of carbohydrate per kilogram per day dependent on their primary form of exercise/activity, activity intensity, sex, and environmental conditions [88]. The great variance that exists in the demands of sports, training, and level of play make it difficult to provide a single concise recommendation. As training duration and intensities increase carbohydrate requirements rise. Young athletes lack even the large range recommendations that are provided for adult athletes. The recommendations for young athletes suggest at least 50% of young athletes diet should be in the form of carbohydrate [89] or between 3 and 8 grams [90] of carbohydrate per kilogram of body mass dependent primarily on exercise intensity.

The role of carbohydrate ingestion around active occasions is an area of intense study. Early research into carbohydrate's role in exercise performance examined the effect of blood glucose levels and physiological state following prolonged exercise [91, 92]. Additional research investigated muscle glycogen's role in muscle fatigue [93, 94]. Subsequent research investigated the role of carbohydrate ingestion following exercise to restore muscle glycogen stores following the depletion related to exercise stress [95]. More recently the research focus has shifted to explore the role of carbohydrate ingestion during exercise stress in sustaining exercise intensity and improve performance [96–98].

It is commonly suggested that normal body stores of carbohydrate can be a significant fuel source for approximately 90–120 minutes of moderate to vigorous exercise. While this statement is accurate in many exercise settings where individuals are exercising at moderate-vigorous intensities typically associated with endurance exercise, more detailed investigation would demonstrate exercise intensity is a vital component in understanding glycogen depletion rates. Significant glycogen depletion can occur anywhere from ~10 minutes with supramaximal intensities to greater than 3 hours at low exercise intensities [99]. This oversimplification needs to be considered as we look at the findings from carbohydrate research. Many of the research investigations providing our understanding of carbohydrate needs around exercise are based on endurance type exercise. As we consider the sports typically involving young athletes some of our understanding may not fully translate.

As stated previously part of our limited understanding in the nutritional needs of young athletes is the result of proper research ethics. The research described above with adults involved muscle biopsies, exercise to failure, and exercise resulting in “poor” physiological states. This type of request would be inappropriate to make to children. Therefore most of our understanding of youth athletes comes from the utilization of less invasive techniques. Young athletes have been shown to have a lower respiratory exchange ratio (RER) during exercise at similar relative submaximal intensities (%${\dot{V}\text{O}}_{2\;\max}$) as their adult counterparts [4]. Based on the RER changes resulting from the shift from fat as a primary fuel source to increasing carbohydrate utilization, this would suggest young athletes are better able to utilize fat as a fuel or are potentially limited in their maximal performance as a result of not being able to utilize carbohydrate readily enough at higher intensities.

Research has shown that increasing glycogen stores will enhance exercise performance [100] and reductions in muscle glycogen content correspond with increasing levels of fatigue. Unfortunately, young athletes have been shown to store less glycogen than adults [4]. During prolonged exercise and exercise at elevated intensities reduced glycogen levels will lead to early onsets of fatigue. Due to their lower glycogen stores, young athletes will likely experience accelerated rates of fatigue. This accelerated fatigue is a result of the inability of the body to maintain sufficient blood glucose levels to meet the young athletes elevated glucose needs of the brain as compared to adults [4].

With reduced muscle glycogen stores the need for exogenous sources of carbohydrate becomes increasingly more important in the maintenance of exercise intensity. Much of our understanding regarding the role of carbohydrate ingestion in exercising youth is the result of the work of the researchers in the Children's Exercise and Nutrition Centre at McMaster University. Riddell demonstrated adolescent athletes utilized lower absolute amounts of exogenous glucose as compared to values reported in adults [101]. However, exogenous carbohydrate utilization has been demonstrated as a greater relative contributor to total energy utilization in the young athlete even with lower absolute utilization rates [58, 101].

Current research foci have shifted more towards the improvement of health and wellness as opposed to performance. However recent research continues to demonstrate the ergogenic effects of carbohydrate ingestion on youth sport. Dougherty et al. demonstrated performance improvements with basketball skills test with carbohydrate compared to water ingestion [102]. Batatinha et al. demonstrated gymnasts experienced a reduction in the number of falls from a balance beam with carbohydrate ingestion during a session [103]. Smith et al. demonstrated performance was improved with carbohydrate ingestion during football skill performance [104].

While the need for carbohydrate is recognized as important, the differences between youth and adult are still not fully understood. Research has shown carbohydrate ingestion spares endogenous carbohydrate stores in exercising youth while at the same time youth seem to be unable to utilize carbohydrate at rates similar to those seen in adults [101]. Research in adults has led to the establishment of guidelines suggesting carbohydrate ingestion during activities lasting 45 minutes or longer provides an ergogenic effect with doses varying from “small amounts including mouth rinse” up to 90 g/h [88]. Currently, specific recommendations similar to what exists for adults are not available for the youth athlete.

For performance enhancement young athletes will benefit from the ingestion of carbohydrate during exercise. Without specific rates recommended for the young athletes, we must rely on the recommendations of adults and refine carbohydrate intakes during exercise based on trial and error methods. These recommendations suggest athletes should ingest simple sugars at a rate of 30–60 g/h for exercise lasting longer than 60 minutes. Additionally, athletes should ingest 1–1.5 g/kg of body mass in the 30 minutes following cessation of prolonged exercise [15]. The ingestion of carbohydrate during exercise should be considered an equally significant component of the training plan as the skill aspects of sport.

## 6. Water

As with their adult counterparts hydration status during sport is important to performance in young athletes. It could be argued that, due to their increased susceptibility to succumb to heat stressors, due to their greater body surface areas to body mass ratio, hydration is a more important consideration in young athletes [105]. In addition to an increased body surface area to body mass ratio, adolescents have been shown to have diminished sweat rates as compared to their adult counterparts [106]. Diminished sweat rates are advantageous as a result of their protection of body water status but are disadvantageous due to the reduced ability to dissipate heat. Added importance of hydration is due to the fact that, in addition to performance decrements, hypohydration has been shown to lead to increased physiological strain, increased risk of heat injury/illness, and increased perceived exertion at similar workloads [15, 107].

As a result of hypohydration, the body experience fluid shifts resulting in increased cardiovascular strain as plasma volume declines [108, 109]. Additionally, the impaired cardiovascular function also leads to diminished skin blood flow resulting in a decline in the ability to dissipate heat to the environment [110]. Hypohydration also leads to an increase in the perception of exertion required to maintain a steady work rate. Research investigating the role of hydration on exercise performance has shown fluid ingestion and the maintenance of proper hydration status improve performance [102, 111].

To add to the physiological strain associated with dehydration inherent with physical activity, most athletes have been found to arrive to practice in a hypohydrated state [106, 112]. Even with education emphasizing the need for proper hydration, preactivity hydration assessments of athletes involved in sport have demonstrated significant percentages of the population to be hypohydrated [113]. Kavouras et al. reported that educational interventions significantly improved preexercise hydration status, however following education regarding hydration 66% of the youth athletes reported to practice in a hypohydrated state [114].

While unlikely to be a broad risk in youth sport, attention must also be drawn to the potential to ingest fluids at excessive levels resulting in the risk of hypernatremia and potentially death. Since the first reports in the literature of hyponatremia in endurance events greater focus has continued to grow regarding the risks associated with over drinking [115]. Hyponatremia has been reported to be as high as 51% in ultramarathons performed under hotter ambient temperatures [115]. The incidence of hyponatremia increases as duration of activity increases along with fluid ingestion. A potential means to reduce the rate of plasma sodium concentration decline is through the ingestion of sodium containing beverages [116]. It should be noted that very few beverages actually contain sodium levels sufficient to maintain plasma sodium levels; however the ingestion of sodium containing beverages will reduce the rate of decline.

The American College of Sports Medicine's Position Stand on Nutrition and Athletic Performance recommends athletes to consume 5–7 mL/kg of body mass 4 hours prior to exercise, enough fluid to reduce body mass changes to less than 2% during activity, and 450–675 mL for every 0.5 kg of body mass lost during exercise [15]. These recommendations do not account for age but are likely a good place to begin for youth athletes since they are based on body mass rather than absolute volumes. Hydration goals should be to minimize body mass losses associated with dehydration while ensuring fluid ingestion does not exceed sweat losses. This is easily assessed through body weight measures immediately prior to and immediately following activity. Increases in body mass would inform the athlete fluid ingestion was too high and significant decreases in body mass would inform the athlete fluid ingestion was insufficient. Additionally, in situations where repeated days of exercise are performed in the heat sodium ingestion rates should be increased to maintain plasma sodium levels. Flavored fluids have been repeatedly shown to aid in the maintenance of fluid intake reducing voluntary dehydration.

Equation for determining sweat rate is as follows:

(1) $$\begin{array}{c} \frac{{\text{BW}}_{0}+{\text{DF}}_{0}-{\text{BW}}_{E}-{\text{DF}}_{E}}{\text{Time (hrs)}}, \end{array}$$

where BW_0_ is body mass before exercise, DF_0_ is mass of exercise food and drink before exercise, DF_*E*_ is body mass after exercise, and DF_*E*_ is mass of exercise food and drink after exercise.

## 7. Micronutrients

Micronutrients categorically refer to vitamins and minerals used by the body during normal physiological functions. Generally, it is accepted that a well-balanced diet of sufficient caloric intake will provide the adequate micronutrients to support normal growth and maturation. The American Medical Association (AMA) and the American Dietetic Association (ADA) recommend nutrients be obtained from food sources rather than supplements in healthy children [117]. Likewise, the American Academy of Pediatrics (AAP) does not endorse regular supplementation of vitamins and minerals in healthy children (with the exception of fluoride in unfluoridated areas). However, AAP has noted that some children are at increased risk of nutrient deficiencies. Specifically, AAP suggests that children and adolescents with anorexia or poor appetites, chronic diseases, and food insecurity are at greater risk for nutrient deficiencies. Youth who do not consume adequate amounts of dairy or have sufficient sun exposure may also be at risk for deficiencies [118].

The daily time constraints of an elite young athlete can make achieving a balanced diet difficult, thus putting these individuals at a potential increased risk for micronutrient deficiencies as well. These deficiencies are most commonly observed in girls rather than boys and in mineral intake rather than vitamin intake. Athletic youth may actually be more likely to achieve the recommended intake of vitamins than nonathletic youth due to their increased total caloric intake. Adequate intake of minerals appears to be a bigger challenge for youth, particularly girls [119]. Specifically, iron and calcium are frequently noted as common nutritional concerns among children and adolescents.

Iron deficiency and subsequent anemia are common in adolescents [120]. Increases in hemoglobin production, blood volume, and muscle mass are normal characteristics of growth and maturation and account for the majority of increased iron needs in developing adolescents. However, iron requirements become even greater for girls at the onset of menses. Iron-deficient anemia has significant, negative implications on performance in adults [121, 122] and youth [123–125]. Diminished performance is most apparent in iron-deficient anemic athletes participating in activities with higher aerobic demands (i.e., endurance event athletes) [124]. The recommended intake of iron for boys and girls aged 9–13 years is 18 mg per day. Boys and girls aged 14–18 years should consume 11 mg and 15 mg per day, respectively [126]. In accordance with AMA, ADA, and AAP guidelines, youth should improve their iron status through consumption of iron-rich foods at meals such as red meat, beans, and green vegetables. Athletes will likely find additional benefit by including other iron-rich foods such as peanuts and dried fruits and iron-fortified cereals as regular snacks. Furthermore, the inclusion of foods higher in ascorbic acid with these nonheme iron sources will improve iron absorption from these snacks [127].

Calcium requirements are greatest during adolescence, 1,300 mg per day for both boys and girls [128]. This higher requirement accommodates the prime opportunity for acquisition of bone mass that spans the pubertal ages. Availability of nutrients critical for bone development (e.g., calcium) and opportunity for increased bone loading (e.g., physical activity) prior to achieving skeletal maturation is critical in preventing osteoporosis later in life [129]. Despite the greater requirements and the clear benefits of consuming adequate amounts, United States children and adolescents' average intake falls below the minimum recommendations [130]. In the American diet, the majority of dietary calcium is obtained from milk and other dairy sources [131]. However, milk consumption has shown a general decline in this age group [132]. Given the perishability of most dairy products, young athletes face practicality issues scheduling regular consumption throughout the day. Breakfast consumption is associated with greater calcium intake among this age group [133] and should be strongly encouraged. Likewise, athletes should consume regular snacks that include rich sources of calcium (e.g., fortified orange juice, almonds, and broccoli), throughout the day. Furthermore, calcium absorption is dependent on adequate levels of vitamin D [128]. Thus, attention to sources of calcium fortified with vitamin D is warranted, particularly among individuals who are not likely to be exposed to sufficient sunlight to endogenously produce adequate vitamin D. While whole food sources are always preferred, the convenience of calcium supplements (often fortified with vitamin D) may make adequate intake a more viable possibility.

An additional consideration warranting attention in young athletes participating in large amounts of training and competition is the potential need to replenish sodium and potassium lost in sweat. The summation of the electrolyte loss resulting from sweat loss has been shown to be equivalent to daily intakes, even in young athletes [105]. Much of this additional loss in salt can be offset through the ingestion of sport drinks during practice and competition, which also partially addresses hydration concerns in the group.

## 8. Summary

Research regarding the nutritional needs of young competitive athletes is sparse and is primarily composed of investigations of youth-adult differences. In addition to the limited research, the majority of our current knowledge in the adult population is based on differences between typical adults compared to their more active counterparts. Research to date suggests similarities in the caloric and macronutrient needs of active adults and their younger counterparts; however, youth-adult differences in fuel utilization have also been clearly demonstrated. In addition to the energy needs of highly active youth, nutritional intake plays a critical role in the growth and development of young athletes and should be a principal emphasis at this stage in their lives. General guidelines for nutrition for active youth are summarized in Table 2. These guidelines serve as recommendations that support healthy growth and development and also account for the additional caloric needs of active youth. As emphasis on performance outcome goals continues to increase in youth athletics, active youth quickly become young athletes. Likewise, nutritional considerations move beyond increased caloric needs and promotion of healthy growth and development to nutritional strategies that can optimize performance. A summary of available performance-based nutritional strategies can be found in Table 3. The paucity of data examining the unique needs of young athletes draws attention to increased need for further investigations on this subject which consider the distinct nutritional requirements of growth and maturation at all age and skill levels. Given the popularity of youth sports and the increasing demand for performance outcomes, increased attention on this topic is warranted by the scientific community.

## Figures and Tables

**Table 1:** Age-specific energy requirements for boys and girls who participate in heavy physical activity levels.

Age	Boys	Girls
(years)	(kcals/day)	(kcals/day)
6-7	1,800	1,650
7-8	1,950	1,775
8-9	2,100	1,950
9-10	2,275	2,125
10-11	2,475	2,300
11-12	2,700	2,475
12-13	2,925	2,625
13-14	3,175	2,725
14-15	3,450	2,855
15-16	3,650	2,875
16-17	3,825	2,875
17-18	3,925	2,875

Adapted from FAO/WHO/UNU, 2004 [7].

**Table 2:** General nutrition recommendations for maintaining health.

Protein	15–20% of total calories should come from protein; 0.8–1.2 g/kg/day derived from whole food sources

Fat	>15% and <30% of total calories should come from fat

Carbohydrate	>50% of total calories should come from carbohydrates, or 3–8 g/kg/day

Micronutrients	Regular supplementation is not recommended in healthy children and adolescents consuming a balanced diet

**Table 3:** Supplemental nutrition recommendations for athletes.

Protein	1.2–1.8 g/kg/day derived from whole food sources
	*After exercise*: 20 g of high quality protein shortly after exercise

Carbohydrate	*During exercise*: 30–60 g/hr for exercise lasting more than 1 hour
	*After exercise*: 1.0–1.5 g/kg of body mass within 30 minutes of exercise cessation

Fluid	*Before exercise*: 5–7 mL/kg 4 hrs prior to exercise
	*During exercise*: assess sweat rate and develop hydration plan to maintain body mass during exercise
	*After exercise*: 450–675 mL/0.5 kg and additional sodium consideration to account for loss through sweat

Micronutrients	*During exercise*: sodium to offset losses associated with sweat being lost in sweat
