# Erratum to “Nutritional Considerations for Performance in Young Athletes”

**DOI:** 10.1155/2017/6904048

**Published:** 2017-11-22

**Authors:** JohnEric W. Smith, Megan E. Holmes, Matthew J. McAllister

**Affiliations:** Department of Kinesiology, Mississippi State University, P.O. Box 6186, Mississippi State, MS 39762, USA

In the article titled “Nutritional Considerations for Performance in Young Athletes” [1], there was an error in the breakdown of equation (1), where both body mass and food and drink mass are called out to use the same symbol. Accordingly, “DF_*E*_ is body mass after exercise, and DF_*E*_ is mass of exercise food and drink after exercise” should be corrected to “BW_*E*_ is body mass after exercise, and DF_*E*_ is mass of exercise food and drink after exercise.”
